# Fulminant hepatitis secondary to dengue in pediatric patients: a series of four cases

**DOI:** 10.1590/S1678-9946202668015

**Published:** 2026-02-16

**Authors:** Isabelly Victoria Simões Melchiori, Mariana Favero Elias, Jhonny Richard de Sousa, Leonardo Labbate Kalil, João Pedro de Mattos Anacleto, Victor Fernandes Rosa, Ana Cristina Aoun Tannuri

**Affiliations:** 1 Universidade de São Paulo, Faculdade de Medicina, Hospital das Clínicas, São Paulo, São Paulo, Brazil; 2 Universidade de São Paulo, Faculdade de Medicina, Hospital das Clínicas, Departamento de Cirurgia Pediátrica, São Paulo, São Paulo, Brazil

**Keywords:** Acute liver failure, Dengue, Liver transplantation, Severe dengue; Pediatrics

## Abstract

This study reports a case series of four pediatric patients with dengue who developed fulminant hepatitis and acute liver failure requiring transplantation. This study aims to enhance early recognition of dengue-related complications, particularly fulminant hepatitis. The series includes two previously healthy girls, aged one and six years; and two boys, aged 12 and 15 years, with pre-existing hemoglobinopathies. A review was conducted to contextualize these cases with prior reports of dengue-associated fulminant hepatitis in children, focusing on diagnosis and management. All four patients underwent liver transplantation, yet their clinical courses and outcomes were varied. Patient 1, a six-year-old girl, had early warning signs and underwent cadaveric liver transplantation nine days after onset. Patient 2, a one--year-old girl, developed severe disease and received living-donor transplantation 20 days after onset. Both had favorable postoperative outcomes. Patient 3, a 12-year-old boy with SC hemoglobinopathy, underwent transplantation on day seven of illness but died on the fifth postoperative day. Patient 4, a 15-year-old boy with sickle cell anemia, underwent transplantation on day four of symptoms but suffered multiple cardiorespiratory arrests during recovery and died on postoperative day 24. Although rare, dengue can lead to fulminant hepatitis in previously healthy children, with worse outcomes in those with comorbidities. Early recognition of severe manifestations is critical for appropriate management. Larger studies are warranted to identify prognostic factors and optimize decision-making for pediatric patients requiring liver transplantation.

## INTRODUCTION

Dengue is an arboviral disease endemic to tropical and subtropical regions, caused by any of the four dengue virus serotypes (DENV-1, DENV-2, DENV-3, and DENV-4), belonging to the *Flaviviridae* family and transmitted mainly by the *Aedes aegypti* mosquito.

Brazil has the highest number of dengue cases worldwide^1^. The year 2024 was historic for dengue in the country, with a record 1,345,801 probable cases reported^2^. This trend continued into 2025, with over 1.2 million cases and 1,420 confirmed dengue-related deaths reported between January and May^3^. However, the actual number of infections is certainly higher. The estimate is that the proportion of asymptomatic infections is 59%, increasing up to 65% during outbreak periods^4^. The disease usually follows a self-limiting course when symptomatic, with spontaneous resolution, especially in patients without comorbidities or social risk conditions.

The clinical presentation of dengue is highly variable, ranging from asymptomatic infection to mild cases—with fever, headache, retro-orbital pain, myalgia, rash, pruritus, and gastrointestinal symptoms such as vomiting, abdominal pain, and diarrhea—or severe forms. Warning signs include intense abdominal pain, persistent vomiting, bleeding, lethargy, hepatomegaly, and evidence of plasma leakage, such as ascites, pleural effusion, and increased hematocrit. In younger children, particularly infants, the signs may be nonspecific, making differential diagnosis with other febrile viral illnesses challenging^5,6^. Brazilian protocols recommend case stratification based on the presence of warning or severity signs to guide appropriate clinical management.

Although most cases follow a benign course, severe complications can arise, particularly during the critical phase of the disease. Among the less frequent manifestations, acute hepatic dysfunction has gained attention as a potentially fatal complication. Elevated transaminases are common laboratory finding, but in rarer cases, dengue may progress to fulminant hepatitis—characterized by massive hepatocellular necrosis and rapid onset of hepatic encephalopathy and coagulopathy. Although the incidence of acute liver failure in severe dengue is low (0.31%–1.1%), the associated mortality is high (20%–68.3%)^7,8^. Histopathological findings in livers of children with fatal dengue have shown necrosis, vascular congestion, and inflammatory infiltrates, in addition to viral antigen detection and inflammatory mediators, reinforcing the role of immune response and direct viral injury in the pathogenesis of fulminant hepatitis^9^.

Despite the severity of this outcome, the association between dengue and fulminant hepatic failure remains underreported in the scientific literature, mainly documented through case reports and clinical series. Reports of cases requiring liver transplantation are even more scarce, with little evidence of prognostic factors. The rarity of this condition, combined with the need for early intervention and management in specialized centers, underscores the importance of systematic discussion and reporting of such cases.

The cases in this article contribute to the literature by describing pediatric patients and comparing the clinical course of four individuals—two previously healthy and two with hemoglobinopathies—who rapidly developed severe hepatic dysfunction secondary to dengue infection, culminating in the need for liver transplantation. Moreover, the different outcomes highlight the association between pre-existing comorbidities and negative prognosis, as both comorbid patients died during the same hospitalization. Such occurrences emphasize dengue as an atypical but possible etiology of fulminant hepatitis in children.

## MATERIALS AND METHODS

This study is a case report series, based on the clinical evaluation, medical records, and surgical descriptions of four patients: two previously healthy female patients, aged one and six years; and two male patients, aged 12 and 15 years, both with pre-existing hemoglobinopathies. In addition to the case descriptions, searches were performed in medical databases (PUBMED, LILACS) for reviews and case reports on dengue complications, particularly the progression to fulminant hepatitis, including aspects of diagnosis and treatment.

### Ethics

The study was approved by the Universidade de Sao Paulo, Faculdade de Medicina, Hospital das Clinicas institutional Ethics Committee, protocol N° 5835324.

## CASE 1

Age: 6 years and 8 months (current); 6 years and 2 months (at transplantation).

Sex: female.

Comorbidities: asthma.

### Clinical history

The patient's symptoms began on December 22, 2024, with fever, vomiting, and prostration. She was evaluated at an urgent care unit, where dengue was diagnosed (positive NS1 test), and was discharged with clinical guidance.

On December 26, she returned to the unit with worsening general condition and laboratory findings indicating impaired liver function. At that time, bullous lesions were also observed on the lower limbs, prompting transfer to a tertiary hospital.

Upon admission to the intensive care unit (ICU), supportive measures and antibiotic therapy with ceftriaxone were initiated. The clinical condition of the patient worsened, with signs of plasma leakage. An ultrasound performed on December 28 revealed a liver at the upper limit of normal size, periportal edema, splenomegaly, a small right pleural effusion, a laminar left effusion, and free fluid in the abdominal cavity.

On the same day, her consciousness level declined and she developed hemodynamic instability, requiring orotracheal intubation, mechanical ventilation, and escalation of antibiotic therapy. The pediatric surgery team was consulted due to progressive worsening of liver function.

The patient was transferred and admitted to the Instituto da Crianca e do Adolescente (ICR) of the Universidade de Sao Paulo, Faculdade de Medicina, Brazil, on December 29, with severe liver failure and an indication for liver transplantation. Laboratory tests revealed markedly elevated liver enzymes, with an ALT exceeding 4,000 U/L, accompanied by a total bilirubin of 6.03 mg/dL, an INR of 5.97, and a serum albumin level of 2.7 g/dL (Table 1).

**Table 1:** Laboratory and imaging tests.

Case 1
Laboratory findings (12/29) -Complete blood count: Hemoglobin 8.9 / Platelets 20,000 / Leukocytes 55,610 (58% neutrophils, 21% lymphocytes) -Liver function: Total bilirubin 6.03 (direct 3.29, indirect 2.74) / GGT 220 / ALP 630 / Fibrinogen 40 / INR 5.97 / Total protein 5.0 / Albumin 2.7 / Ammonia 314 / AST 911 / ALT 4412 -Venous blood gas: pH 7.24 / pO_2_ 55 / pCO_2_ 37 / HCO_3_ 15.9 -Other tests: CRP 65.9 / Ferritin >33,511.2
**Case 2**
Laboratory findings (02/17) -Complete blood count: Hb 12 / Ht 35.1% / WBC 8930 (Neutrophils 41.2%, Lymphocytes 52%) / Platelets 84,000 -Liver function: Total protein 6.4 / Albumin 3.4 / Globulin 3 / ALP 563 / GGT 132 / AST 2059 / ALT 1442 / Total bilirubin 18 (direct 14.2, indirect 3.8) / PT 9 / INR 7.75 / aPTT 63.3s (ratio 2.14) / Fibrinogen 109 -Venous blood gas: pH 7.38 / pCO_2_ 22 / pO_2_ 69 / BE -10 / HCO_3_ 13 / O_2_ sat 93% -Other tests: CRP 1 / Venous lactate >135
**Case 3**
Laboratory findings (03/09/25) -Complete blood count (CBC): Hemoglobin 5.9 / Hematocrit 15.3 / Leukocytes: 61,570 (Neutrophils 65%, Lymphocytes 15%, Left shift up to myeloblasts) / Platelets 75,000 -Renal function and electrolytes: Urea 124 / Creatinine 3.96 / Sodium 133 / Potassium 7.1 / Chloride 95.2 -Liver function: Total proteins 7.1 / Albumin 4.1 / aPTT ratio 3.9 / INR >10 (uncoagulable) / Fibrinogen 106 / AST >7,000 / ALT >7,000 / GGT 30 / ALP 318 / Total bilirubin 11.35 / Direct bilirubin 3.7 / Indirect bilirubin 7.65 / Ammonia 771 -Venous blood gas analysis: pH 7.18 / pCO_2_ 36 / pO_2_ 36 / BE -15 / HCO_3_ 13 / Lactate 125 -Other serologies: Negative.
Laboratory findings (03/15/25) -Complete blood count (CBC): Hb 8.5 / Ht 25.4 / Leukocytes 17,640 (Neutrophils 66%, Lymphocytes 19%, Monocytes 3%) / Platelets 11,000 -Renal function and electrolytes: Urea 98.5 / Creatinine 3.24 / Sodium 132 / Potassium 6.7 / Chloride 96 -Liver function: AST 6565 / ALT 1515 / GGT 136 / ALP 990 / Total bilirubin 11.25 / Direct bilirubin 5.86 / Indirect bilirubin 5.39 / Ammonia 183 / CRP 51.5 / Total proteins 3.5 / Albumin 2 / INR 7.85 / Fibrinogen 106 -Venous blood gas analysis: pH 7.21 / pCO_2_ 34 / pO_2_ 47 / BE -14.3 / HCO_3_ 13.6 / Lactate 121
**Case 4**
Laboratory findings (03/16) -Complete blood count (CBC): Hemoglobin 4.3 / Hematocrit 11.5% / Leukocytes 30,920 (Neutrophils 59%, Lymphocytes 24%) / Platelets 41,000 -Renal function and electrolytes: Creatinine 1.36 / Urea 73 / Chloride 103 / Calcium 10.2 / Sodium 137 / Potassium 7.3 -Liver function: Total bilirubin 14.41 / Direct bilirubin 7.24 / Total proteins 5.4 / Albumin 3.3 / ALP 373 / AST 19,974 / ALT 5,416 / aPTT ratio 2.45 -Others: D-dimer 19,423 ng/mL / CRP 115 -Serologies: Negative. CMV and EBV: IgG reactive, IgM non-reactive, PCR negative for both. Dengue: NS1 negative, IgM and IgG reactive. Imaging - Ultrasound (03/16): Hepatomegaly with periportal edema and relative increase in arterial vascularization. Although nonspecific, these findings are consistent with acute hepatitis.
Laboratory findings (04/14) -Complete blood count (CBC): Hemoglobin 6.7 / Hematocrit 17.4 / Leukocytes 8,560 / Platelets 7,000 -Liver function: Total proteins 4.6 / Albumin 2 / Fibrinogen 222 / AST 202 / ALT 79 / GGT 20 / ALP 173 / Total bilirubin 39 (Direct 21 / Indirect 18) / Ammonia 119 -Venous blood gas analysis: pH 7.02 / pCO_2_ 40 / pO_2_ 45 / HCO_3_ 10.3 / BE -20.7 / Lactate > 180 -Others: CRP 103

On admission, she was pale, icteric, and her heart rate was 136 beats/min, besides a prolonged capillary refill, weak pulses, requiring norepinephrine, vasopressin, and dobutamine, on mechanical ventilation, with diffuse bleeding from venipuncture sites and oral mucosa, bullous lesions on lower limbs, and mild palpebral and peripheral edema.

A functional echocardiogram showed an ejection fraction of 85%, enabling suspension of dobutamine. New cultures were collected, and antibiotic therapy was maintained with vancomycin, cefotaxime, and clindamycin. Dialysis was performed on December 30 due to high ammonia levels, in addition to transfusion of red blood cells and fresh frozen plasma.

On December 31, she underwent liver transplantation with a reduced-size cadaveric donor graft (male, 24 years old), with cold ischemia time of 4h50min and warm ischemia time of 45 minutes. During the procedure, she received blood components (packed red blood cells, fresh plasma, fibrinogen) and vasopressors (norepinephrine and vasopressin). In the immediate postoperative period, vasopressin was discontinued due to hemodynamic stability. Norepinephrine was discontinued the next day. She remained on ventilatory and dialysis support. She was extubated on January 7, transferred from ICU to ward on January 15, and discharged from hospital on February 4. She remains under outpatient follow-up.

## CASE 2

Age: 2 years and 6 months (current); 1 year (at transplantation).

Sex: female.

### Clinical history

The clinical course of the patient began on January 31, 2024, with complaints of dysuria and foul-smelling urine. On February 4, she developed petechial lesions on the lower limbs, and on February 5, dengue was confirmed (positive IgM serology), with thrombocytopenia (platelets: 92,000/mm^3^). She received treatment for a urinary tract infection (UTI) with ceftriaxone for six days and was later discharged with cephalexin. However, on February 14, she developed worsening jaundice (orange-colored urine), decreased oral intake, and neurological deterioration, with alternating episodes of somnolence and irritability. She was again taken to the urgent care unit on February 17, where a sepsis protocol was initiated with fluid resuscitation and ceftriaxone administration. She was admitted to the ICU, where severe liver failure was confirmed via laboratory findings (Table 1) associated with clinical hepatic encephalopathy.

The patient was transferred once more due to probable need for liver transplantation. On February 18, she had neurological deterioration, with no response to painful stimuli, involuntary chewing movements, and limb hypertonia, leading to orotracheal intubation (IOT) for neuroprotection.

On February 20, she underwent a living-donor liver transplantation, with her maternal aunt as the donor, due to the severity of liver failure. She received blood components (fresh plasma, platelets, fibrinogen) and balanced crystalloid solutions. Intraoperatively, she experienced significant bleeding, requiring escalation of norepinephrine, addition of vasopressin, and adrenaline infusion.

In the immediate postoperative period, the patient remained hemodynamically stable, without signs of clinical deterioration. She was extubated on February 22, showing favorable recovery and progressive improvement in general condition. During hospitalization, she experienced episodes of seizures, managed by the neurology team. She was transferred to the ward on March 3 and discharged on March 15. During outpatient follow-up, she had multiple episodes of acute rejection and incisional hernia, as well as infectious complications related to immunosuppression, with hospitalizations for meningitis and acute otitis media, both associated with breakthrough seizures.

## CASE 3

Age: 12 years (at diagnosis and transplantation).

Sex: male.

Past medical history: hemoglobinopathy SC.

### Clinical history

This patient developed symptoms on March 5, 2025, with abdominal pain, vomiting, poor appetite, myalgia, and prostration. On March 7, he sought urgent care, where the NS1 antigen test for dengue was positive. He was discharged with symptomatic treatment and azithromycin. The following day, he returned in poor general condition, febrile, somnolent, with signs of hemodynamic instability, weak pulses, delayed capillary refill, and cold extremities. Initial management included fluid resuscitation with lactated Ringer's solution, ceftriaxone (50 mg/kg), opioid analgesia, and vasoactive drug support. Laboratory tests showed impaired liver and kidney function, coagulation disorders, and thrombocytopenia (32,000). He was admitted to the pediatric intensive care unit (PICU), where he progressed with decreased level of consciousness and hypotension. Orotracheal intubation, central venous catheter placement, and invasive arterial pressure monitoring were performed without complications.

During his PICU stay, he received platelet transfusion (17 ml/kg), plasma (10 ml/kg), vitamin K, calcium, and albumin. Antibiotic therapy was escalated to cefepime, and symptomatic medications were administered. Cranial CT was performed due to decreased consciousness, which revealed no abnormalities. The patient developed severe metabolic acidosis, acute kidney injury (creatinine clearance of 40 ml/min/1.73 m^2^), oligoanuria, and fulminant hepatitis. Dialysis was initiated due to refractory acidosis and hyperammonemia, but it was not tolerated because of hypotension, requiring interruption of the procedure and blood reinfusion.

He was then registered in the CROSS system (Central Regulation of Health Services) for referral to a liver transplant center. He was admitted to the PICU at ICR, on March 9. At admission, he was on mechanical ventilation, hemodynamically unstable, and requiring high doses of vasoactive drugs. Initial laboratory tests revealed an INR greater than 10, as well as AST and ALT levels exceeding 7,000 U/L, and a total bilirubin of 11.35 mg/dL (Table 1).

On March 12, a cadaveric donor liver transplantation was performed (graft weight: 750 g), with biliary reconstruction via Roux-en-Y enteroenterostomy. Cold ischemia time was five hours, and warm ischemia one hour. Intraoperatively, there was uncontrolled bleeding due to the poor general condition and underlying disease of the patient, with estimated blood loss exceeding 500 mL. He received fresh frozen plasma, platelets, and 12 units of packed red blood cells.

He returned to the PICU in poor general condition, hemodynamically unstable, with persistent renal dysfunction (Table 1) manifested by oligoanuria and resistance to furosemide. Renal replacement therapy was initiated the following day with initial tolerance but repeatedly had to be suspended due to hypotension.

Despite volume resuscitation, high-dose vasoactive drugs, and suspension of dialysis, the patient remained hypotensive throughout the night of March 16. On the morning of March 17, he had mid-dilated fixed pupils, and sedation was withdrawn without evidence of supraspinal reactivity. Later that morning, he developed bradycardia followed by cardiac arrest, and death was declared.

## CASE 4

Age: 15 years (at diagnosis and transplantation).

Sex: male.

Past medical history: sickle cell disease.

## CLINICAL HISTORY

A patient with sickle cell disease was admitted to the emergency department on March 16 with fever (up to 38.9 °C, two daily peaks), dark-colored urine with reduced volume, prostration, pallor, and oxygen desaturation at home (SpO_2_ 75%). On admission, he was somnolent, icteric, with marked mucocutaneous pallor, respiratory distress, hepatomegaly, and diffuse abdominal pain without peritoneal signs. He was immediately taken to the resuscitation area and received fluid resuscitation, one unit of packed red blood cells, and ceftriaxone, with partial improvement. Laboratory tests showed a marked increase in liver enzymes (Table 1), raising the hypothesis of fulminant hepatitis. Etiologic investigation revealed reactive dengue IgM and IgG.

Admitted to the ICU, he developed nocturnal desaturation and decreased level of consciousness, requiring orotracheal intubation. During the procedure, he had black, foul-smelling emesis; a nasogastric tube was placed, and epistaxis with dark clots was noted.

Given the markedly elevated ammonia (800 μg/dL) and hyperkalemia, continuous hemodialysis (CRRT; PRISMA) was initiated. He progressed with hypotension, requiring escalating doses of norepinephrine, subsequent addition of vasopressin, and a hydrocortisone loading dose.

On March 18, he developed desaturation and reduced right-sided chest expansion. Chest radiography showed a right pleural effusion, confirmed by lung POCUS, without indication for drainage. Echocardiography revealed concentric left ventricular hypertrophy and a small pericardial effusion. The following day he remained stable, tolerated dialysis, and vasoactive agents were weaned.

He underwent deceased-donor liver transplantation on March 20 (51-year-old donor, whole liver). Intraoperatively, he received seven units of packed red blood cells, two units of plasma, and one unit of platelets.

Postoperatively, he had persistently elevated bilirubin, high output from surgical drains, hyperammonemia, and refractory hypervolemia, requiring continuous dialysis and parenteral nutrition. His respiratory status improved, enabling extubation on March 29, with stability on nasal cannula. However, he continued to show significant neurological impairment (made eye contact but without interaction or response to commands).

On April 4, exploratory laparotomy was performed for suspected retained clot, revealing a perihepatic and hilar hematoma. Thorough cavity irrigation was undertaken; the biliary anastomosis was intact and the hepatic artery patent.

He remained hemodynamically stable off vasoactive agents, on high-flow nasal cannula, with continuous dialysis and progressive reduction of drain output. On April 7, he developed a 10-minute cardiorespiratory arrest due to respiratory failure and hypoxemia; return of spontaneous circulation was achieved after intubation and resuscitation. He evolved with bilateral mydriasis and a seizure controlled with phenytoin. A new cardiac arrest occurred on April 13 (six minutes), also reversed. After this event, he became hemodynamically unstable, requiring increasing norepinephrine doses and the addition of vasopressin. Antimicrobial coverage was broadened with discontinuation of vancomycin and initiation of tigecycline per peritoneal lavage culture.

On April 14, the patient developed refractory shock, with hyperlactatemia and worsening liver function (Table 1).

He progressed to asystole, and given the irreversible condition, no further resuscitative efforts were undertaken according to prior agreement with the family. Death was declared on postoperative day 24 after liver transplantation.

## DISCUSSION

Although most dengue cases follow a benign or even asymptomatic course, the cases reported here highlight the potential of dengue virus infection to trigger fulminant hepatitis requiring liver transplantation, including in previously healthy pediatric patients. Patients Number 1 and Number 2, aged six years and one year, respectively, developed acute liver failure in distinct contexts, without any prior history of liver disease, because of dengue confirmed by serology. Conversely, Patients Number 3 and Number 4 had previous comorbidities and, despite the early recognition of warning signs, died after liver transplantation. The history of hemoglobinopathy in both may have contributed to the unfavorable clinical course and poor outcome.

Both cases 1 and 2 emphasize not only the rare presentation of dengue as a trigger for fulminant hepatitis in children, but also the need for early vigilance for warning signs and hepatic involvement. Indicating transplantation in infectious contexts such as dengue remains challenging, particularly in the pediatric population, requiring careful evaluation of hepatic function and neurological status^10^.

The similarities between cases 3 and 4, with both patients dying during the same hospitalization, raise questions regarding the association between pre-existing hematological disease and a worse prognosis, whether in terms of hemodynamic instability at the time locations or mortality. Few studies^11^ in international literature address fulminant hepatitis in pediatric patients with dengue, and even fewer report cases undergoing transplantation. Teerasarntipan *et al*.^12^ sought predictors of acute liver failure in patients with dengue-induced hepatitis and observed that pre-existing liver comorbidities, high INR levels, and low serum albumin were associated with increased risk of developing liver failure (OR 3.8, 95%CI 1.1–13.9, p < 0.05). However, these comorbidities did not increase the risk of mortality, and the association in transplanted cases was not studied.

The decision to proceed with liver transplantation in the third patient, despite his extremely critical preoperative condition, was guided by the absence of alternative therapeutic options and the rapid deterioration of hepatic function. In pediatric patients, liver transplantation for fulminant hepatic failure is frequently undertaken under extremely severe conditions, often including hemodynamic instability during surgery^13^. Nevertheless, children possess a remarkable physiological reserve and capacity for recovery, which enables many to stabilize once hepatic function is restored after transplantation. Our institution is the national reference center with the largest experience in pediatric liver transplantation for fulminant hepatitis in Brazil, and several recipients with similar preoperative instability have achieved full recovery after graft implantation. Thus, although the decision to operate in such circumstances is difficult and carries a substantial risk, it is guided by the potential for reversibility observed in pediatric patients, in whom liver replacement can rapidly reestablish metabolic and hemodynamic equilibrium.

The post-transplant course in the cases that evolved well and were discharged, particularly in the case of Patient 2, reinforces the importance of long-term follow-up, considering specific complications such as rejection, infections, and neurological disorders. Factors such as the recipient's preoperative condition, donor type, and timing of hepatic deterioration directly influence prognosis. In cases Number 3 and 4, the presence of comorbidities may have been related to the unfavorable outcomes, emphasizing the importance of heightened attention to early signs of clinical deterioration in patients with comorbidities.

Therefore, the aforementioned cases contribute to the recognition of dengue as an emerging cause of liver failure in children and reinforce the need for well-established protocols for early identification, severity stratification, and referral to centers capable of performing pediatric liver transplantation, in addition to the need for meticulous follow-up during and after hospitalization.

## CONCLUSION

Given the cases that progressed within a short period to severe acute liver failure secondary to dengue virus infection, it is essential to emphasize the importance of measures ranging from prevention to advanced therapeutic management, including liver transplantation. During the febrile phase of the disease, proper guidance regarding hydration, rest, and identification of warning signs is crucial, as these measures may prevent progression to severe forms. These signs must be recognized early—including by patients and families themselves—to ensure prompt medical care. In the presence of severe acute liver failure, the patient is given priority on the transplant list, a scenario that demands rapid and specialized action from the surgical team. Although transplantation represents a chance of survival, pre-existing conditions may contribute to poorer outcomes, but larger studies are needed for statistical correlation. Even in cases with good evolution, after hospital discharge, patients remain vulnerable to complications, requiring rigorous and prolonged outpatient follow-up.

## Data Availability

The complete anonymized dataset supporting the findings of this study is included within the article itself.
